# Binary Bamboo Forest Growth Optimization Algorithm for Feature Selection Problem

**DOI:** 10.3390/e25020314

**Published:** 2023-02-08

**Authors:** Jeng-Shyang Pan, Longkang Yue, Shu-Chuan Chu, Pei Hu, Bin Yan, Hongmei Yang

**Affiliations:** 1 College of Computer Science and Engineering, Shandong University of Science and Technology, Qingdao 266590, China; 2 Department of Information Management, Chaoyang University of Technology, Taichung 41349, Taiwan; 3 College of Electronic and Information Engineering, Shandong University of Science and Technology, Qingdao 266590, China

**Keywords:** bamboo forest growth optimization, binary, optimization, transfer function, feature selection

## Abstract

Inspired by the bamboo growth process, Chu et al. proposed the Bamboo Forest Growth Optimization (BFGO) algorithm. It incorporates bamboo whip extension and bamboo shoot growth into the optimization process. It can be applied very well to classical engineering problems. However, binary values can only take 0 or 1, and for some binary optimization problems, the standard BFGO is not applicable. This paper firstly proposes a binary version of BFGO, called BBFGO. By analyzing the search space of BFGO under binary conditions, the new curve V-shaped and Taper-shaped transfer function for converting continuous values into binary BFGO is proposed for the first time. A long-mutation strategy with a new mutation approach is presented to solve the algorithmic stagnation problem. Binary BFGO and the long-mutation strategy with a new mutation are tested on 23 benchmark test functions. The experimental results show that binary BFGO achieves better results in solving the optimal values and convergence speed, and the variation strategy can significantly enhance the algorithm’s performance. In terms of application, 12 data sets derived from the UCI machine learning repository are selected for feature-selection implementation and compared with the transfer functions used by BGWO-a, BPSO-TVMS and BQUATRE, which demonstrates binary BFGO algorithm’s potential to explore the attribute space and choose the most significant features for classification issues.

## 1. Introduction

Nowadays, with the rapid growth of the computer industry, a wide variety of data has been affected. The high speed of development has led to a discontinuous growth in the dimensionality and sample size of the data collected. Managing these data is becoming increasingly difficult. In the early stages of computing, attempts were made to manage these data sets using manual management, but as features in the data set increased, this approach became impractical [1,2]. With further developments, data mining and machine learning application techniques have been developed. Practical applications such as statistical analysis, neural networks and pattern recognition also have surfaced [3,4,5,6]. However, the data collected are often accompanied by high noise levels, mainly caused by the immaturity of the technology used to collect the data and the provenance of the data themselves. There is no doubt that extracting useful content and patterns from such large and noisy data is an extremely challenging task [7].

Feature selection (FS) is an effective method for reducing dimensionality and removing noisy and unreliable data. The aim is to remove unnecessary features from the whole feature set and finally obtain a representative subset [8]. FS is very important and essential for data scientists and machine learning practitioners. A good feature-selection method can simplify models, improve learning accuracy, reduce runtime and help understand the underlying structure of the data, which can significantly influence further improved models and algorithms. A high-quality sample is key to training a classifier. The performance of a classifier is directly influenced by the presence of redundant or irrelevant features in the sample [9].

A realistic data set is usually represented by a collection of data containing plenty of features, not all of which are useful for classification. Redundant, irrelevant features can reduce classification accuracy. As the dimensionality of the data rises and the search space expands, selecting the best subset of features becomes increasingly challenging. In general, the enumeration method cannot solve the problem of finding the optimal subset of features, so some strategies are needed to find the subset of features, and the popular search strategies are global search, heuristic search and random search [10,11,12]. Although existing search techniques have achieved good results in feature selection, there is still a high probability of slipping into a local optimum. Therefore, to solve the feature selection-problem more effectively, a direct and effective search strategy is needed.

The heuristic optimization algorithm is a common optimization method to solve optimization problems. It has a high search power and search speed for NP problems, which can obtain a better solution in polynomial time [13,14]. It solves feature selection by converting successive optimization algorithms into binary versions using transfer functions (tfs).

Heuristic algorithms are inspired by nature, social behavior or the behavior of groups of organisms [15]. It puts forward feasible solutions to optimization problems by imitating natural phenomena and biological behaviors, but the quality of solutions is very different. The original heuristics suffered from the following problems: they rely too much on information about the organization of the algorithm, have low applicability and can easily slip into a local optimum solution. With the development of heuristics, meta-heuristics have emerged that are different from the original heuristics, adding the idea of random search and possessing generality compared to traditional heuristics. Although the meta-heuristic algorithm is improved compared with the original heuristic algorithm, neither is guaranteed to obtain an optimal global solution, and due to the addition of the idea of random search, repeated executions may converge to a globally optimal solution. There are four main categories of meta-heuristic algorithms based on the type of inspiration: evolution-based algorithms, group intelligence-based algorithms, human-based algorithms, physics and chemistry-based algorithms [16]. The main inspiration for evolution-based algorithms comes from the evolutionary law of survival of the fittest (Darwin’s law).

Genetic algorithms (GA) [17,18,19] are the main representatives, such as differential evolution (DE) [20,21,22] and quasi-affine transformation evolution (QUATRE) [23,24,25]. Population intelligence optimization algorithms simulate group intelligence to reach a globally optimal solution. Each group in the algorithm represents a population of organisms that, through the cooperative behaviour of group members, can accomplish tasks that are impossible for individuals. Examples include Particle Swarm Optimization (PSO) [26], Cat Swarm Optimization (CSO) [27], Fish Migration Optimization (FMO) [28], Whale Optimization Algorithm (WOA) [29,30,31], etc. Human behavior, including teaching, social, learning, emotional and managerial behavior, is a major source of inspiration for human-based algorithms. Examples include teaching and learning-based optimization (TLBO) [32], social learning optimization (SLO) [33], social-based algorithms (SBA) [34], etc. Physical rules and chemical reflections in the universe inspire physics and chemistry-based algorithms. Examples include simulated annealing (SA) [35], gravitational local search (GLSA) [36], etc.

Nonetheless, discrete problems are always popular among optimization problems, such as feature selection and shop floor scheduling problems. Continuous optimization algorithms are not suitable for solving such problems, so there is a need to convert continuous optimization algorithms into discrete versions. So far, scholars have proposed many binary versions of algorithms applied to feature selection, while many scholars have also implemented improvements to existing binary algorithms and achieved better results. For example, the classical PSO, GWO and PIO algorithms have been successfully applied to feature selection. Hu et al. improved BGWO by introducing a new transfer function to replace the S-shaped function. Then, a new parametric equation and an improved transfer function were proposed to improve the quality of the solution [37]. Tian et al. analysed BPIO, introduced four new transfer functions along with an improved velocity update equation, and successfully implemented feature selection with better results [38]. Liu et al. devised an improved multi-swarm PSO (MSPSO) to solve the feature-selection problem while combining SVM with F-score methods to improve generalization [39]. However, many metaheuristics have been redesigned without consideration of the problem of sliding into local optima.

Bamboo Forest Growth Optimization (BFGO) is a meta-heuristic algorithm inspired by the bamboo growth process, recently proposed by Chu et al. It is applied to wireless sensor networks (WSNs) and has been effective in reducing energy consumption and improving network performance [40]. This research aims to propose a BFGO with a binary version for the application of discrete optimization problems such as FS. This paper converts the algorithm to a binary version using transfer functions, the better-known ones being the S−type transfer function family and the V−type transfer function family [41]. A novel type of transfer function is also introduced: the Taper-shaped transfer function [42]. The 23 benchmark functions test and compare the performance of different types of transfer functions. The evaluation of the Binary Bamboo Forest Growth Optimization (BBFGO) algorithm against cutting-edge, sophisticated and efficient algorithms shows that the proposed BBFGO possesses optimal or sub-optimal solutions to the problem of finding optimal values. The main contributions are as follows.

1. The first binary bamboo forest growth optimization algorithm (BBFGO) is proposed.
2. Based on a mathematical analysis approach, the first analysis is carried out for the search space of binary BFGO. Based on the results of this analysis, the V−transfer function is stretched in two ways, two new curvature V−transfer functions for binary BFGO are proposed and the new curvature V−transfer function is successfully verified to have better performance in the test function.
3. The long-mutation strategy is introduced to the original BBFGO to avoid solution stagnation, and a new mutation approach is proposed.
4. BBFGO and BBFGO with the new mutation method are compared in test functions with advanced algorithms, and it is confirmed that the long-mutation strategy of the new mutation method improves the performance of BBFGO. Compared with the advanced algorithm, the new mutation strategy leads BBFGO to complete the reversal.
5. BBFGO is applied to feature selection and compared with cutting-edge algorithms, which performs well in low and high dimensional classification accuracy. In particular, it is more competitive on high-dimensional data sets.

The paper is organized as follows: Section 2 introduces bamboo forest growth optimization, Section 3 presents a concrete implementation of binary bamboo forest growth optimization based on mathematical analysis, proposing three classes of transfer functions, BBFGO-S, BBFGO-V, BBFGO-T, and introducing a novel mutation approach to prevent the optimization process from stalling. Section 4 shows the experimental results of the families of BBFGO transfer functions compared to BPSO-TVMS, BGWO-a and BQUATRE and the effect of ABBFGO on the performance improvement. Section 5, ABBFGO-S, ABBFGO-V and ABBFGO-T algorithms are used for feature selection and compared with the three advanced algorithms in Section 4.

## 2. Bamboo Forest Growth Optimization

**Figure 1:**
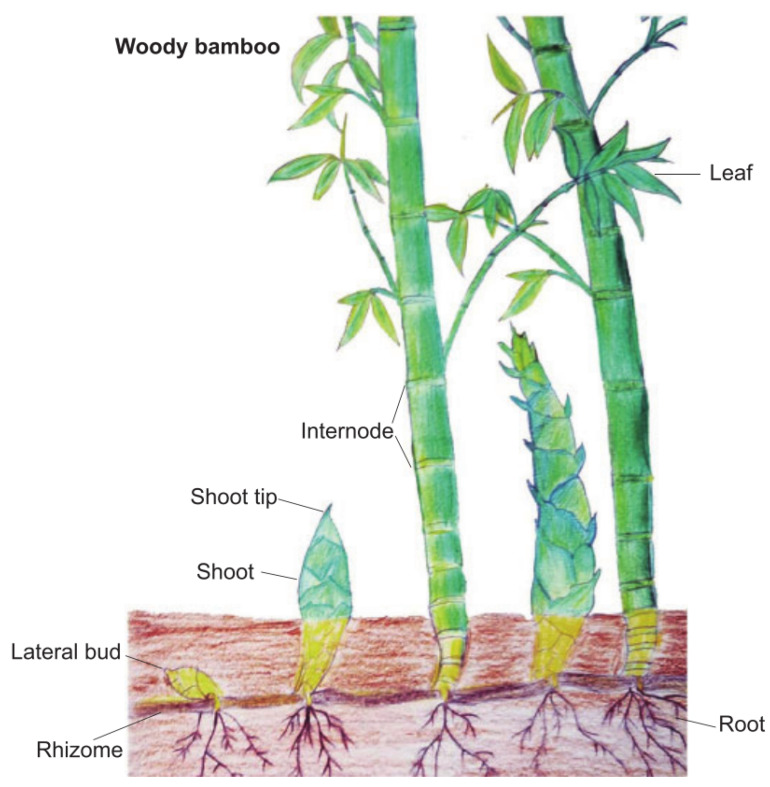
The structure of bamboo.

FS is an effective method for dimensionality reduction of data, which is widely used and plays an important role in machine learning and pattern recognition. By reducing the dimensionality of the data set, the computational speed of the model is improved. This section focuses on bamboo forest growth optimization. Bamboo is a fast-growing herb and one of the world’s fastest-growing plants. Bamboo has underground rhizomes, also known as bamboo whips, which grow horizontally and produce roots on the nodes called whip roots. Each node has a shoot that has the opportunity to grow into a new whip or bamboo shoot. The new bamboo whip will continue to spread underground, and the bamboo shoot will break through the soil and develop into a bamboo pole, and then gradually develop into a bamboo forest. Figure 1 shows the specific structure of the bamboo. Bamboo whip plays an important role in the overall growth of bamboo forests, and it expands the living area of bamboo and provides nutrients for the growth of bamboo. According to Guihua Jin [43], bamboo has unique growth characteristics compared to other grasses because the tall stems of bamboo grow rapidly within 2–3 months with a slow and slow growth rhythm. This trait may help them adapt to the environment and stand out from the competition to survive. The growth of a bamboo forest can be divided into two stages: (a) bamboo whip extension; (b) bamboo shoot growth. In addition, a bamboo forest can correspond to more than one bamboo whip, and a bamboo whip can only belong to one bamboo.

Recently, Chu and Feng et al. proposed a novel optimization method inspired by the growth behavior of bamboo forests: bamboo forest growth optimization (BFGO). It views the global extension of the bamboo whip as the development phase of the algorithm and the growth of bamboo shoots as the exploration phase of the algorithm, where the shoots emerge through the soil to become bamboo shoots, and the emerged bamboo shoots have only a small probability of growing into bamboo.

1. Extension of the Bamboo Whip

**Figure 2:**
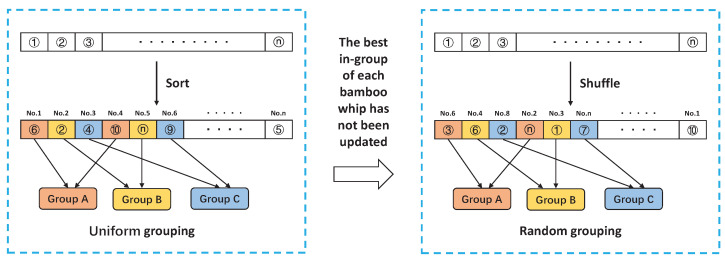
The idea of meme grouping.

Based on the relationship between bamboo forest and bamboo whip, the concept of clustering is added to the algorithm. While optimizing the algorithm, individuals are grouped by memes, and dynamic adjustment is made between uniform grouping and random grouping. The uniform grouping is based on individual fitness, and the fitness is arranged in descending order. All the individuals in the initial population are arranged from high to low according to the fitness function value to form a sequence, and then the meme group is classified to divide the bamboo forest into multiple meme groups evenly. Random grouping is used when the renewal of the best individuals of the bamboo whip within each group has all stalled and will re-break up the individuals for random assignment. The idea of meme grouping is shown in Figure 2.

The direction of the extension of the bamboo whip underground is influenced by three factors: group cognition, bamboo whips memory and bamboo forest centre, which correspond to global optimal, intra-group optimal and bamboo forest centre, respectively. The formula for the centre position is shown in Equation (1). The formula for the extension direction is shown in Equations (2)–(4).

(1) $$\vec{C(k)}=\frac{1}{n}\sum_{i=1}^{n}\vec{X(k_{i})}$$

(2) $$\cos\left(\alpha \right)=\frac{\vec{X_{t}}\cdot \vec{X_{G}}}{\left|\vec{X_{t}}\right|\times \left|\vec{X_{G}}\right|}$$

(3) $$\cos\left(\beta \right)=\frac{\vec{X_{t}}\cdot \vec{X_{P(k)}}}{\left|\vec{X_{t}}\right|\times \left|\vec{X_{P(k)}}\right|}$$

(4) $$\cos\left(\gamma \right)=\frac{\vec{X_{t}}\cdot \vec{C(k)}}{\left|\vec{X_{t}}\right|\times \left|\vec{C(k)}\right|}$$

where $X(k_{i})$ represents the ith bamboo shoot position on the k bamboo whip, $\vec{X_{t}}$ is the current bamboo shoot position, $\vec{X_{G}}$ is the global optimal bamboo shoot position, $\vec{X_{p}\left(k\right)}$ is the optimal bamboo shoot position on the k bamboo whip and C(k) is the central position of the bamboo forest. Moreover, cos(α), cos(β) and cos(γ) represent the degree of extension of the current bamboo shoot position to $\vec{X_{G}}$, $\vec{X_{p}\left(k\right)}$ and C(k), respectively. The formula for the update is shown in Equation (5).

(5) $$X_{t+1}=\left\{\begin{array}{cc} X_{G}+Q\times \left(c_{1}\times X_{G}-X_{t}\right)\times \cos\left(\alpha \right),rand\leq 0.4 & \\ X_{p(k)}+Q\times \left(c_{1}\times X_{p(k)}-X_{t}\right)\times \cos\left(\beta \right),0.4<rand\leq 0.8 & \\ C\left(k\right)+Q\times (c_{1}\times C\left(k\right)-X_{t})\times \cos\left(\gamma \right),else & \end{array}\right.$$

(6) $$Q=2\times (1-\frac{t}{T}),$$

where *Q* is a crucial parameter impacting the step size of the algorithm development and steadily reduces from 2 to 0 as the number of iterations grows, *t* is the current iteration, and *T* indicates the maximum number of iterations; $c_{1}$ is a random number from 0 to 1. Taking a random number and comparing it with 0.4 and 0.8 to determine the direction of extension of the next generation of solutions ensures the diversity of solutions and enhances the algorithm’s ability to find the best.

2. Shoot Growth of the Bamboo

As we all know, the growth of trees is inevitably affected by many random factors. During the whole growth process, these factors have a large and small impact on them, both individually and comprehensively. At present, it is not possible to accurately determine all of them. Even if they can be measured, the relationship between the factors is also random. Therefore, when describing the tree growth process, the tree-measuring factors are generally regarded as random variables, and the tree growth process is described as a random process. Due to the interference of random factors and the different site conditions, the cumulative growth amount of different bamboo at a specific time t is randomly changed, which shows that the growth process of bamboo is random. Based on the characteristics of bamboo shoot growth, Shi et al. constructed a stochastic process model of bamboo shoot growth by using stochastic process theory and the Sloboda growth equation [44]. There are two stages to the growth process: the slow growth stage and the fast growth stage. Combined with this model, the bamboo shoot growth stages grow as shown in Equation (7).

(7) $$X\left(t\right)=X_{G}\times e^{\frac{b}{\omega \times e^{\omega }}}$$

**Figure 3:**
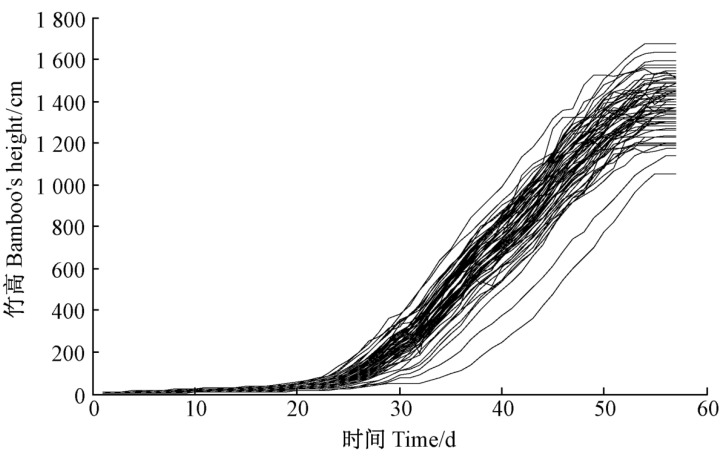
Growth curve of bamboo shoots.

The shape of the bamboo growth increment model is shown in Figure 3. The high growth of the bamboo shoot stage is completed in about 55 days, and the growth of the bamboo shoot stage can be divided into 2 stages around the 25th day: 1–25 days is the first stage, in which the growth of bamboo is relatively gentle, 25–55 is the second stage, in which the bamboo shows explosive growth. Moreover, $X_{G}$ represents the maximum bamboo height in a particular growth environment, varying with the growth environment; b is the bamboo measurement factor, a random variable; and ω is the shape parameter of the model, independent of the environmental conditions.

According to the incremental calculation of changes at different times, the calculation equation is shown in Equation (8). In the multi-dimensional case, the result of Equation (8) is a vector.

(8) $$\Delta H=\frac{X(t)-X(t-1)}{X_{G}-C\left(k\right)+1},$$

where ΔH represents the relationship between the increment between the two generations and $X_{G}$ and C(k), the denominator represents the distance between $X_{G}$ and C(k), and X(t) indicates the cumulative length of the bamboo growth within the t-th generation.

The individual renewal of bamboo shoots at this stage is shown in Equations (9) and (10). In the multi-dimensional case, the result of Equation (10) is a vector.

(9) $$X_{temp}=\left\{\begin{array}{cc} X_{t}+X_{D}\times \Delta H,rand<0.5 & \\ X_{t}-X_{D}\times \Delta H,else & \end{array}\right.$$

(10) $$X_{D}=1-\left|\frac{X_{t}-C\left(k\right)}{X_{G}-C\left(k\right)+1}\right|,$$

where $X_{D}$ represents the ratio relationship between C(k) and the distance between $X_{t}$ and $X_{G}$, which varies to a large extent in the early stages of exploration and stabilizes or even remains constant in the later stages of exploration as $X_{t}$ converges extremely closely to $X_{G}$ and C(k). This results in a more extensive exploration in the early stages and a slow growth towards convergence in the later stages.

In Equation (9) ‘+’ means the distance increases and ‘−’ means the distance decreases, increasing the capacity to search for the optimal solution by expanding the search range and balancing global exploitation and local exploration. The pseudocode of BFGO is shown in Algorithm 1.
**Algorithm 1** Pseudocode of BFGO  1:Initialize the parameters of BFGO: *N* (number of search agents), *T* (maximum iteration), *n* (number of bamboo shoots) and *K* (number of bamboo whips)  2:Initialize the positions of the search agents  3:Calculate the fitness of each search agent  4:The search agents are sorted in descending order of fitness and divided into *K* groups according to the uniform grouping in Figure 2  5:Update $X_{G}$, $X_{P(k)}$, $f(X_{G})$ and $f(X_{P(k)})$  6:t=2  7:**while**t<T+1**do**  8:   **for** each bamboo whip **do**  9:     Update C(k) using Equation (1)10:     Update the $X_{t+1}$ using Equations (2)–(6)11:     Calculate $f(X_{t+1})$12:   **end for**13:   Update $X_{G}$, $X_{P(k)}$, $f(X_{G})$ and $f(X_{P(k)})$14:   **for** each bamboo whip **do**15:     Update C(k) using Equation (1)16:     Update the $X_{temp}$ using Equations (7)–(10)17:     Calculate $f(X_{temp})$18:   **end for**19:   Update $X_{G}$, $X_{P(k)}$, $f(X_{G})$ and $f(X_{P(k)})$20:   t=t+121:   Repeat step 422:**end while**

## 3. Binary Bamboo Forest Growth Optimization Algorithms

In bamboo forest optimization, bamboo constantly changes its position in space. In some special problems, such as feature selection, the solution is limited to binary 0, 1 values, which inspires a special version of BBFGO. BFGO is a novel algorithm for population evolution by updating positional information through optimal global directions, intra-group optimal directions and central direction guides. BFGO incorporates the idea of clustering to achieve co-competition between multiple groups and has a stronger merit-seeking capability compared to other meta-heuristics. The mechanism for converting continuous BFGO to binary BFGO is explained in Section 3.1. The advanced binary BFGO framework with integrated long-mutations is presented in Section 3.2.

### 3.1. Binary Bamboo Forest Growth Optimization (BBFGO)

**Figure 4:**
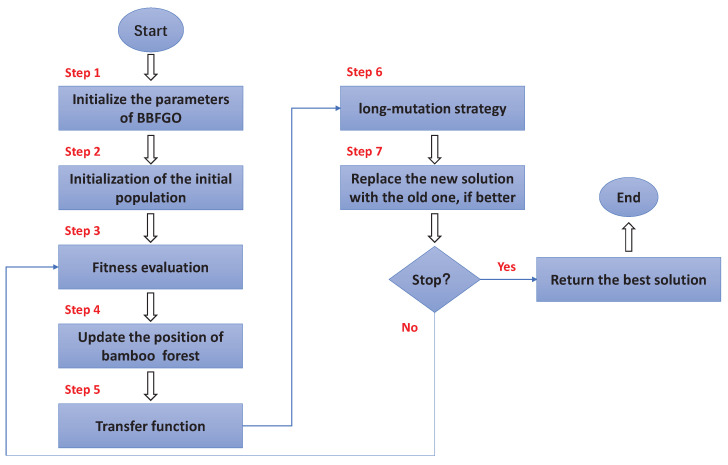
The graphical interpretation of BBFGO.

The standard bamboo forest growth optimization algorithm has continuous solutions and can update the equations without restriction constraints, but for feature selection, the search space needs to be set up as a hypercube, which means that the elements of each solution need to be integrated as 0 or 1. The graphical interpretation of BBFGO is shown in Figure 4.

#### 3.1.1. Mathematical Analysis

Under the constraints of the binary condition, the positions of the bamboo whips and shoots cannot be moved arbitrarily in space, so it is necessary to consider the position structures that belong only to the binary BFGO. For the sake of a simple description of the mathematical model, only the one-dimensional case is considered after analyzing the range of values of the individual parameters. From Equation (6), Q ∈ (0, 2), and from Equations (1)–(3), cos(α), cos(β) and cos(γ) are all ∈ (0, 1), and $c_{1}$ is a random number between 0 and 1, so $c_{1}$ ∈ (0, 1).

In the bamboo whip extension stage, the globally optimal extension direction in Equation (4) is first analysed. Since $X_{t}$ and $X_{G}$ only take 0 or 1, there are four occurrences of calculating the next generation position, and the value of $X_{t+1}$ is calculated as follows.

(1) if $X_{G}$ = 0 and $X_{t}$ = 0

$X_{t+1}$ = $X_{G}$ + Q × ($c_{1}$ × $X_{G}$ − $X_{t}$) × cos(α) = 0

(2) if $X_{G}$ = 0 and $X_{t}$ = 1

$X_{t+1}$ = $X_{G}$ + Q × ($c_{1}$ × $X_{G}$ − $X_{t}$) × cos(α) = Q × (−1) × cos(α)

since Q ∈ (0, 2), cos(α) ∈ (0, 1), then $X_{t+1}$ = (−Q × cos(α)) ∈ (−2, 0).

(3) if $X_{G}$ = 1 and $X_{t}$ = 0

$X_{t+1}$ = $X_{G}$ + Q × ($c_{1}$ × $X_{G}$ − $X_{t}$) × cos(α) = 1 + (Q × $c_{1}$ × cos(α))

since Q ∈ (0, 2), cos(α) ∈ (0, 1), $c_{1}$ ∈ (0, 1), then $X_{t+1}$ = 1 + (Q × $c_{1}$ × cos(α)) ∈ (1, 3).

(4) if $X_{G}$ = 1 and $X_{t}$ = 1

$X_{t+1}$ = $X_{G}$ + Q × ($c_{1}$ × $X_{G}$ − $X_{t}$) × cos(α) = 1 + (Q × ($c_{1}$ − 1) × cos(α))

since Q ∈ (0, 2), cos(α) ∈ (0, 1), $c_{1}$ ∈ (0, 1), then $X_{t+1}$ = 1 + (Q × ($c_{1}$ − 1) × cos(α)) ∈ (−1, 1).

From the above analysis, it can be obtained that $X_{t+1}$ ∈ (−2, 3). Similarly, when calculating the optimal extension direction within the group, as the constituent elements of the $X_{p(k)}$ solution are only 0 or 1, then the analysis method is the same as above, and $X_{t+1}$ ∈ (−2, 3) is obtained. When calculating the central extension direction, the formula for C(k) is given by Equation (1), where $X(k_{i})$ is composed of 0 or 1, then the final result of C(k) ∈ (0, 1), which is brought into the analysis process, the final result is $X_{t+1}$ ∈ (−2, 3). In summary, it can be concluded that $X_{t+1}$ ∈ (−2, 3).

To avoid the search agent missing better solutions due to too large a step, the step size of the exploration in the bamboo shoot growth phase is therefore limited. The accumulation at a specific moment is restricted in Equation (7) to the interval [0, 1].

Since $X_{t}$ takes values only 0 and 1 and ΔH ∈ [0, 1] and $X_{D}$ ∈ (0, 1), in the one-dimensional case there are two occurrences and the value of $X_{temp}$ is calculated as follows:

(1) if $X_{t}$ = 0

$X_{temp}$ = $X_{t}$ ± $X_{D}$ × ΔH = 0 ± $X_{D}$ × ΔH

since ΔH ∈ [0, 1], $X_{D}$ ∈ (0, 1), then $X_{temp}$ ∈ (−1, 1).

(2) if $X_{t}$ = 1

$X_{temp}$ = $X_{t}$ ± $X_{D}$ × ΔH = 1 ± $X_{D}$ × ΔH

since ΔH ∈ [0, 1], $X_{D}$ ∈ (0, 1), then $X_{temp}$ ∈ (−2, 2).

Through the analysis of the two stages, it can be concluded that $X_{t+1}$ ∈ (−2, 3) and $X_{temp}$ ∈ (−2, 2). The final results will be used for further discussion of the transfer function.

#### 3.1.2. BBFGO with Transfer Functions

In BFGO, the solutions represented by the bamboo whip and the bamboo shoot are both continuous values, however, FS is a binary optimization problem where the continuous solution needs to be converted to a discrete solution. The transfer function is one of the dominant methods for solving this type of problem. Transfer functions can map continuous values to the interval 0–1 and then update to binary values of 0 or 1 depending on the probability.

In this study, seven tfs are used for this conversion task. Of these seven tfs, two belong to the S-shaped, three to the V-shaped and two to the Taper-shaped classes. The key job of these tfs is to determine the probability of updating the value of an element to 1 or 0. In FS, the solution consists of either 0 or 1. The pseudocode of BBFGO is shown in Algorithm 2. The main three classes of tfs are described as follows:

**S-shaped transfer function (S-tf):**

**Figure 5:**
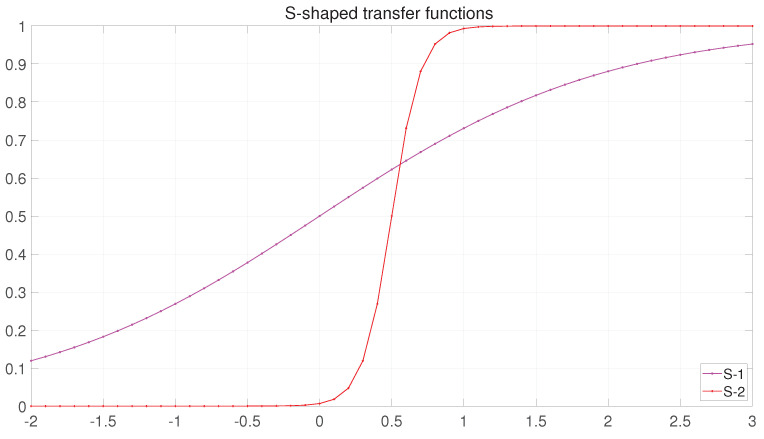
The curves of S-shaped transfer functions.

The curves of the original sigmoid function and another variant are shown in Figure 5. The transfer vector (S) is calculated according to S-shaped using Equations (11)–(13).

(11) $$S_{1}\left(X_{m,j}^{t}\right)=\frac{1}{1+e^{-X_{m,j}^{t}}}\;\forall j=\left(1,2,\ldots ,d\right)$$

(12) $$S_{2}\left(X_{m,j}^{t}\right)=\frac{1}{1+e^{-10*(X_{m,j}^{t}-0.5)}}$$

After obtaining $X_{t+1}$ by Equation (5), it is updated through Equations (11)–(13). Similarly, $X_{temp}$, which is obtained from Equation (9), is updated by Equations (11)–(13).

(13) $$X_{m,j}^{t}=\left\{\begin{array}{cc} 1 & \;\mathrm{rand}\;<S_{i}\left(X_{m,j}^{t}\right),\\ 0 & \;\mathrm{rand}\;\geq S_{i}\left(X_{m,j}^{t}\right). \end{array}\;i=\left(1,2\right)\right.$$

where $X_{m,j}^{t}$ is the element of the dth dimension in the ith solution and $S_{i}\left(X_{m,j}^{t}\right)$ is the probability value of the S-tf based on the mapping of the element of the dth dimension in the ith solution. Whether the final element takes 0 or 1 is determined by Equation (13), with rand being a random value between 0 and 1.

**V-shaped transfer function (V-tf):**

**Figure 6:**
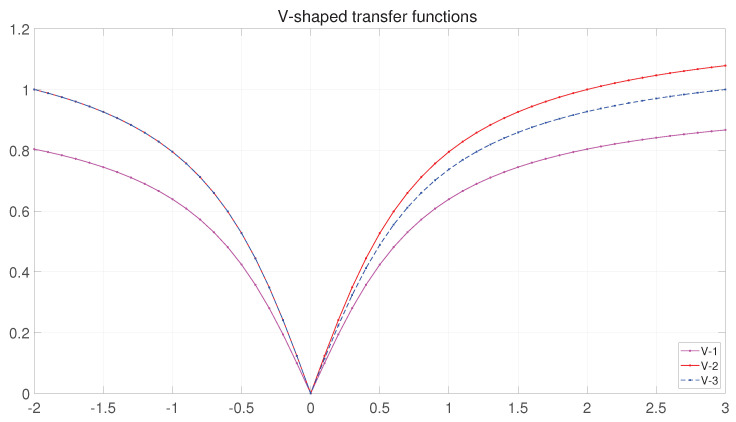
The curves of V-shaped transfer functions.

The curves of the original V-tf and the two variants are shown in Figure 6. The transfer vector (V) is calculated according to V-shaped using Equations (14)–(16):

From the curve V−1 in Figure 6, it can be seen that in the binary BFGO, the bounded maxima of $V_{1}\left(X_{m,j}^{t}\right)$ in the interval [−2, 3] are 0.8038, and 0.8669, respectively, indicating that, even when the bounded values of −2 or 3 of the search space of the search agent are reached, there are still probabilities of 0.1962 and 0.1331, respectively, that 1 is not reached. This contradicts our initial aim to make the search agent’s value large when its large probability of its becoming 1 contradicts this. To resolve this situation, a method of stretching the transfer function is adopted. Since the interval is not symmetric, two approaches to stretching are taken.

(14) $$V_{1}\left(X_{m,j}^{t}\right)=\left|\frac{2}{\pi }atan\left(\frac{\pi }{2}*X_{m,j}^{t}\right)\right|$$

(15) $$V_{2}\left(X_{m,j}^{t}\right)=\left|\frac{atan(\frac{\pi }{2}*X_{m,j}^{t})}{atan(\pi )}\right|$$

(16) $$V_{3}\left(X_{m,j}^{t}\right)=\left\{\begin{array}{cc} \left|\frac{atan(\frac{\pi }{2}*X_{m,j}^{t})}{atan(\pi )}\right| & X_{m,j}^{t}<0\\ \left|\frac{atan(\frac{\pi }{2}*X_{m,j}^{t})}{atan(\frac{3\pi }{2})}\right| & X_{m,j}^{t}\geq 0 \end{array}\right.$$

(17) $$X_{m,j}^{t}=\left\{\begin{array}{cc} 1 & \;\mathrm{rand}\;<V_{i}\left(X_{m,j}^{t}\right),\\ 0 & \;\mathrm{rand}\;\geq V_{i}\left(X_{m,j}^{t}\right). \end{array}\;i=\left(1,2,3\right)\right.$$

where $V_{i}\left(X_{m,j}^{t}\right)$ is the probability value of the V-tf based on the mapping of the element of the dth dimension in the ith solution. The elements take the value of 0 or 1 as determined by Equation (17).

**Taper-shaped transfer function(T-tf):**

The T-tf is a novel transfer function, a primary function constructed from a power function. Its uniform formula is shown in the Equation (18).

(18) $$T\left(x\right)={\left(|\frac{x}{A}|\right)}^{\frac{1}{n}},x\in \left[-A,A\right],n\geq 1$$

where A is a positive real number and n can determine the curvature of the function.

Since the function curve resembles the taper’s tip, it is called the Taper-shaped transfer function. The T-tf has a beneficial effect on the execution time of the elemental discretization process compared to the S-tf consisting of an exponential function and the V-tf consisting of a trigonometric or inverse trigonometric function.

**Figure 7:**
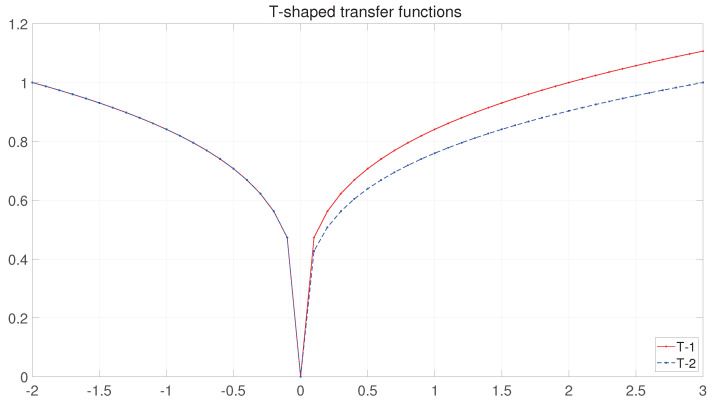
The curves of Taper-shaped transfer functions.

Since the search space of the T-tf is determined by A in Equation (18), however, the analysis in Section 3 shows that the search space of the binary BFGO is a non-symmetric interval, being [−2, 3]. Therefore two different curvature Taper-shaped transfer functions (T-tfs) are proposed. The curves are shown in Figure 7. The transfer vector (T) is calculated according to Taper-shaped using Equations (19) and (20):

(19) $$T_{1}\left(X_{m,j}^{t}\right)=\frac{\sqrt[{4}]{\left|X_{m,j}^{t}\right|}}{\sqrt[{4}]{2}}$$

(20) $$T_{2}\left(X_{m,j}^{t}\right)=\left\{\begin{array}{cc} \frac{\sqrt[{4}]{\left|X_{m,j}^{t}\right|}}{\sqrt[{4}]{2}} & X_{m,j}^{t}<0\\ \frac{\sqrt[{4}]{\left|X_{m,j}^{t}\right|}}{\sqrt[{4}]{3}} & X_{m,j}^{t}\geq 0 \end{array}\right.$$

(21) $$X_{m,j}^{t}=\left\{\begin{array}{cc} 1 & \;\mathrm{rand}\;<T_{i}\left(X_{m,j}^{t}\right),\\ 0 & \;\mathrm{rand}\;\geq T_{i}\left(X_{m,j}^{t}\right). \end{array}\;i=\left(1,2\right)\right.$$

where $T_{i}\left(X_{m,j}^{t}\right)$ is the probability value of the T-tf based on the mapping of the element of the dth dimension in the ith solution. The elements take the value of 0 or 1 as determined by Equation (21).
**Algorithm 2** Pseudocode of BBFGO  1:Initialize the parameters of BFGO: *N* (number of search agents), *T* (maximum iteration), *n* (number of bamboo shoots) and *K* (number of bamboo whips)  2:Initialize the positions of the search agents  3:Calculate the fitness of each search agent  4:The search agents are sorted in descending order of fitness and divided into *K* groups according to the uniform grouping in Figure 2  5:Update $X_{G}$, $X_{P(k)}$, $f(X_{G})$ and $f(X_{P(k)})$  6:t=2  7:**while**t<T+1**do**  8:   **for** each bamboo whip **do**  9:     Update C(k) using Equation (1)10:     Update the $X_{t+1}$ using Equations (2)–(6)11:     Calculate the position under binary conditions of each search agent by Equations (11)–(21)12:     Calculate $f(X_{t+1})$13:   **end for**14:   Update $X_{G}$, $X_{P(k)}$, $f(X_{G})$ and $f(X_{P(k)})$15:   **for** each bamboo whip **do**16:     Update C(k) using Equation (1)17:     Update the $X_{temp}$ using Equations (7)–(10)18:     Calculate the position under binary conditions of each search agent by Equations (11)–(21)19:     Calculate $f(X_{temp})$20:   **end for**21:   Update $X_{G}$, $X_{P(k)}$, $f(X_{G})$ and $f(X_{P(k)})$22:   t=t+123:   Repeat step 424:**end while**

### 3.2. Advanced Binary Bamboo Forest Growth Optimization (ABBFGO)

When a solution is good enough, it will attract other search agents, who will quickly converge towards that solution position. However, there are many local optima in the optimization process. Once BBFGO is stuck in a local trap, all search agents are exploited within a narrow region. The group’s diversity for this feature is discarded, and the best solution is not updated for a while. To break out of the local trap, advanced BFGO (ABBFGO) uses a long-mutation strategy to avoid the algorithm entering stagnation.

Long-mutations, similar to variations in genetic algorithms, are added to improve the global search. The mutation is an important component of evolutionary algorithms because it prevents populations from losing diversity and ensures that wide search space is covered. The long-mutation differs from the short-mutation in that the short-mutation randomly selects a dimension of the solution to mutate, whereas the long-mutation mutates every dimension of the solution.

In this, a new mutation strategy is proposed, which uses a strategy influenced by elites to change the position of the bamboo. The elites are divided into historical elites and contemporary elites, where the historical elites consist of the best solutions within the historical group, and the best solutions within the group are saved to the historical elites when the algorithm iterates to update the global optimum, and the contemporary elites consist of the global optimum and the best solutions within the group in the most recent iteration. When the number of iterations exceeds 10 and the global optimum does not change within three generations, the long-mutation strategy is used to try to escape the trap.

In feature selection, 1 indicates that the feature is used and 0 indicates that the feature is not used. Feature selection aims to achieve high accuracy in classification while selecting as few features as possible. So the solution consists of either 0 or 1. The pseudocode of ABBFGO is shown in Algorithm 3. The long-mutation process considers the influence of contemporary and historical elites on mutation, so each elite type produces one solution, and the following strategy is used when mutating each dimension.

(22) $$X_{-}A_{1}\left(d\right)=\left\{\begin{array}{cc} 0 & \;\mathrm{if}\;\mathrm{rand}\;<\emptyset^{d}\\ 1 & \;\mathrm{else}\; \end{array}\right.$$

where $X_{-}A_{1}\left(d\right)$ indicates the element in the d-dimension of the long-mutation generating solution. $\emptyset^{d}$ is the rate at which the feature is not selected in the d-dimension of the historical elite pool.

(23) $$X_{-}A_{2}\left(d\right)=\left\{\begin{array}{c} \phi_{i}^{d}\;\;\;\;\;\mathrm{if}\;\phi_{i}^{d}=\phi_{j}^{d}\\ \phi_{i}^{d}\;\;\;\;\;\mathrm{else}\;\mathrm{if}\;rand<0.5\\ \phi_{j}^{d}\;\;\;\;\;\mathrm{else}\; \end{array}\right.$$

where $X_{-}A_{2}\left(d\right)$ indicates the element in the d-dimension of the long-mutation generating solution. $\phi_{i}^{d}$,$\phi_{j}^{d}$ is two randomly selected elements in dimension d of the contemporary elite pool whose values are either 0 or 1.
**Algorithm 3** Pseudocode of ABBFGO  1:Initialize the parameters of BFGO: *N* (number of search agents), *T* (maximum iteration), *n* (number of bamboo shoots) and *K* (number of bamboo whips)  2:Initialize the positions of the search agents  3:Calculate the fitness of each search agent  4:The search agents are sorted in descending order of fitness and divided into *K* groups according to the uniform grouping in Figure 2  5:Update $X_{G}$, $X_{P(k)}$, $f(X_{G})$ and $f(X_{P(k)})$  6:t=2  7:**while**t<T+1**do**  8:   **if** t>10&&Convergence(t)==Convergence(t-3) **then**  9:     Use the long-mutation strategy to generate new solutions10:     If the fitness value of the new solution is greater than the global optimal value, replace it11:     Update $X_{G}$, $X_{P(k)}$, $f(X_{G})$ and $f(X_{P(k)})$12:   **end if**13:   **for** i = 1;i ≤ k;i++ **do**14:     **if** $X_{P(k)}$ not updated **then**15:        P++16:     **end if**17:   **end for**18:   **if** P = k **then**19:     Do Random grouping20:   **end if**21:   **for** each bamboo whip **do**22:     Update C(k) using Equation (1)23:     Update the $X_{t+1}$ using Equations (2)–(6)24:     Calculate the position under binary conditions of each search agent by Equations (11)–(21)25:     Calculate $f(X_{t+1})$26:   **end for**27:   Update $X_{G}$, $X_{P(k)}$, $f(X_{G})$ and $f(X_{P(k)})$28:   **for** each bamboo whip **do**29:     Update C(k) using Equation (1)30:     Update the $X_{temp}$ using Equations (7)–(10)31:     Calculate the position under binary conditions of each search agent by Equations (11)–(21)32:     Calculate $f(X_{temp})$33:   **end for**34:   Update $X_{G}$, $X_{P(k)}$, $f(X_{G})$ and $f(X_{P(k)})$35:   Convergence(t) = $f(X_{G})$36:   t=t+137:   Repeat step 438:**end while**

## 4. Experimental Results and Analysis

In this section, the simulation experiment process of BBFGO is mainly introduced. The main purpose is to reveal the effects of various transfer functions and stretched transfer functions on the performance of binary BFGO through 23 benchmark test functions. Table 1 describes the basic information of these 23 benchmark functions. Single-peak functions (1–7), multi-peak functions (8–13) and fixed-dimensional functions (14–23) are examples of reference functions. Furthermore, **opt** is the minimum value that the test function can reach in theory; **parameter space** is the search space of the search agent; **Dim** is the dimension of the function.

To validate the results, BBFGO is compared with the original V-tf, the V-tf after two different ways of stretching and the T-tf. Table 2 shows details of the comparison methods. Table 3 shows information about the parameters of the optimization algorithm recommended and used in solving the examples. Each algorithm has a population size of 30. The maximum iteration is 500 times, and the experiment is run 30 times.

### 4.1. Experimental Analysis of the Transfer Functions and ABBFGO

Table 4 shows the results using different transfer function family algorithms and ABBFMO. A total of 23 benchmark test functions can achieve the minimum values shown in the Opt column of Table 1 for a given continuous solution space, however when testing the BBFGO family of algorithms, the solution space is hypercubic, meaning that the solutions all consist of binary values 0 and 1, so the optimum values that can be achieved under these conditions are not the same. The experimental data in Table 2 are marked in red if an algorithm achieves the minimum of the two-value condition or performs best in the test function. Experimental data where ABBFGO is more effective than BBFGO are marked in blue.

The single-peak test function without local traps is used to test the convergence performance of the different algorithms. If an algorithm performs well in the single-peak test function, it can be shown to have a strong convergence exploitation ability. The first seven functions in Table 4 show that the traditional sigmoid transformation function performs the least well in terms of convergence capability. In $F_{1}$, $F_{2}$, $F_{3}$ and $F_{6}$, all except BBFGO reach the theoretical minimum in the binary condition. For $F_{4}$, BBFGO-S and BBFGO-V1 reach the theoretical minimum, and the two stretched V2 and V3 do not outperform V1 in terms of optimal values, but this difference is not significant. In $F_{5}$, all algorithms do not reach the theoretical optimum and are some distance away from the optimum. BBFGO obtains the worst result at 124.4, but V2 and V3 are stronger than V1 in terms of effect. T1 is closest to the optimum at 1.9333. In $F_{7}$, the best result is obtained by the stretched V2, which shows that stretching the transfer function to the interval [0, 1] helps to improve the BFGO-V convergence development capability.

As the multi-peak function has many local optima, it can be used to test the performance of different algorithms to jump out of the local trap. For the $F_{8}-F_{13}$ test functions, BBFGO performs the worst, and other algorithms achieve theoretical minima in $F_{9}$, $F_{10}$, $F_{11}$ and $F_{12}$. It indicates that BBFGO* has better performance in jumping out of local traps. For $F_{8}$ and $F_{13}$, it can be seen that the stretched V2 and V3 outperform V1. It shows that stretching V-tf also decreases the likelihood of the binary BFGO slipping into a local optimum. BBFGO* performs very well in all the fixed-dimensional functions, except T2, which does not search for a theoretical minimum in $F_{23}$. It illustrates two things: firstly, it reflects the excellent performance of BBFGO*. The second is that there is a limit to how much the transfer function can improve BBFGO performance, especially in functions with fewer local minima or lower dimensionality.

ABBFGO beats BBFGO in 12 out of 23 test functions, which are focused on single and multi-peak test functions. The long-mutation method using elite learning can help change the search space in case the algorithm becomes stuck. ABBFGO outperforms BBFGO significantly, demonstrating the effectiveness of the new mutation method.

### 4.2. Experimental Results for Cutting-Edge Algorithms

BQUATRE is a novel binary algorithm inspired by matrix iteration, Binary QUATRE (BQUATRE) is a binary version that can be used to solve binary application problems [46]. Binary Grey Wolf Optimizer (BGWO) extends the application of the GWO algorithm and is applied to binary optimization issues. In BGWO-a, new a-parameters are used to control the values of A and D, the ability to balance global and local search and the use of a new transfer function to improve the quality of the solution [37]. BPSO-TVMS introduces a new time-varying mirror S-shaped transfer function to enhance global exploration and local exploitation in the algorithm [45]. In Table 5, BBFGO and ABBFGO are compared with these novel improved algorithms, where red font indicates that BBFGO and ABBFGO were defeated, green font indicates that BBFGO is defeated but not ABBFGO, and blue font indicates that both BBFGO and ABBFGO won.

As can be seen in Table 5, in the single-peak test function, all the data colors are green except for $F_{4}$, where BBFGO, which had a poor effect, reverses under the long-mutation strategy, further demonstrating the effectiveness of the strategy. The effect of the new mutation in the multi-peak test function is also unquestionable, with only the $F_{10}$ function failing to beat BQUATRE. BBFGO and ABBFGO outperform BPSO-TVMS in $F_{8}$ and $F_{13}$. ABBFGO takes advantage of the fact that, if the strategy falls into a local trap in the update iteration, it will take the direction of the overall elite to learn to jump out of that trap and search for another better value. Although it helps BBFGO improve its capacity to escape from local optima, this strategy has a limit to the improvement. For example, in $F_{10}$ and $F_{11}$, it does not help BBFGO to achieve the search for the theoretical minimum. It also illustrates the importance of the transfer function in another way. It is the reason why this paper focuses on both the transfer function and the strategy at the same time. All of the above algorithms perform well in the fixed-dimensional test functions, with only BGWO-a performing poorly in $F_{19}$ and $F_{20}$.

## 5. Apply to Feature Selection

Dealing with enormous data owing to their size is extremely challenging due to the abundance of noise and unnecessary aspects in data mining, and which features apply to a learning algorithm is unknown, it is essential to pick the pertinent features from the set of features that the learning algorithm will find useful. As a result, the data set’s characteristics must be reduced. The majority of studies focus on techniques with great accuracy and few characteristics. This section uses the wrapper approach to feature selection.

### 5.1. Datasets Description

The data sets were taken from the UCI machine learning repository [47], and the details of these data sets are shown in Table 6. Table 6 shows the main characteristics of these data sets in terms of the number of features, number of instances and number of classes. The selected data sets were categorized by dimensionality as low-dimensional and high-dimensional data sets, which varied in the number of features and instances and could be used as a sample of the many problems tested. The more novel data set details are as follows: the data set of Turkish Music Emotion in Table 6 is designed as a discrete model, and there are four classes in the data set: happy, sad, angry and relaxed; the data set of LSVT Voice Rehabilitation includes standard perturbation analysis methods, wavelet-based features, fundamental frequency-based features and tools used to mine nonlinear time-series; the data set of Musk (Version 1) describes a set of 92 molecules of which 47 are judged by human experts to be musks, and the remaining 45 molecules are judged to be non-musks. The goal is to learn to predict whether new molecules will be musks or non-musks; the data set of Dermatology contains 34 attributes, 33 of which are linear-valued and one of them is nominal, the diseases in this group are psoriasis, seboreic dermatitis, lichen planus, pityriasis rosea, chronic dermatitis and pityriasis rubra pilaris; instances in the data set of Ionosphere are described by two attributes per pulse number, corresponding to the complex values returned by the function resulting from the complex electromagnetic signal.

### 5.2. Simulation Results

Several cutting-edge algorithms are chosen for comparison tests with the proposed binary BFGO algorithms to confirm the performance in various dimensions, and deeply examine the application. By examining the results of the experiments, such as the accuracy and the number of features selected, and fitness function values for specific evaluation criteria, it is possible to compare the merits of the BBFGO* with other algorithms. The benefits of the proposed binary BFGO algorithm and other algorithms may be compared by examining several outcomes from the feature-selection experiment, such as accuracy, number of feature selections and fitness function values for specific evaluation criteria.

#### 5.2.1. KNN and K-Fold Validation

A fundamental and straightforward machine learning method called the K-Nearest Neighbor (KNN) algorithm classifies data by calculating the separation between various eigenvalues. In KNN classification, a classification population is produced from an input learning instance. The category of an object’s neighbours determines its classification. The category given to the object is determined by the K nearest neighbours’ most common classification (K is a positive number, typically smaller). The calculation method is shown in Equation (23).

(24) $$D_{1}\left(x,x^{\prime }\right)={\left(\sum_{k=1}^{m}{\left|x\left(k\right)-x^{\prime }\left(k\right)\right|}^{l}\right)}^{\frac{1}{l}}$$

where l has two values, 1 and 2. When *l* = 1 $D_{1}(x,x^{\prime })$ denotes the Manhattan distance and when *l* = 2 $D_{2}(x,x^{\prime })$ denotes the Euclidean distance. *x* and $x^{\prime }$ are the two input instances for calculating the distance.

In machine learning modelling, to reduce the probability of overfitting problems without adjusting the model parameters in the test data. The original data set is randomly divided into K parts for K-fold cross validation. One of the K parts is utilized as test data, while the remaining K-1 parts are used as training data. The experiment is run K times, and in the end, the average value of the K experimental outcomes is calculated.

#### 5.2.2. Evaluation Criteria

There are many metrics to judge the merits of an algorithm in feature selection, for example, classification accuracy and the number of feature subsets. However, if only classification accuracy is chosen as an evaluation metric, there is no guarantee that the number of subsets is small, so one influence cannot be considered alone. Suitable evaluation metrics need to be constructed to reconcile the balance of factors. In the simulations, the following criteria were used for evaluation.

(25) $$\;\mathrm{Fitness}\;=\alpha \times {K-Fold}_{\left(\mathrm{error}\right)}+\beta \times \frac{\left|\mathrm{NoSF}\right|}{\left|\mathrm{NoAF}\right|}$$

where ${K-Fold}_{\left(\mathrm{error}\right)}$ is the classification error after completing cross-validation. NoSF is the subset feature after feature selection and NoAF is the number of features for the data set. α and β balancing the classification accuracy and the number of subsets, with α being 0.99 and β being 0.01.

#### 5.2.3. Result Analysis

In the analysis of the results of the binary BFGO algorithm, ABBFGO, ABBFGO-S, ABBFGO-V2, ABBFGO-V3, ABBFGO-T1, ABBFGO-T2, BPSO-TVMS, BQUATRE and BGWO-a were compared. They were run 15 times on each selected data set, with 100 iterations each. The population size in each population is 30. The value of K-Fold parameter in cross validation is 10. The K value in KNN is 5, and the error rates corresponding to individuals were calculated using the five-nearest neighbour approach.

As can be seen from Table 7, ABBFGO and ABBFGO-V3 beat all other algorithms once, ABBFGO-V2 beat all other algorithms six times, and ABBFGO-T2 beat all other algorithms four times in completing the classification correctly. ABBFGO-V2 is also at a good level for feature subsets and meets the feature-selection requirements. In Table 8, ABBFGO-V2 has an overall ranking of 20. Collectively ABBFGO-V2 performs best, with the best classification accuracy and feature subsets. A comparison of the classification accuracy of the Advanced binary BFGO algorithm family (ABBFGO*) with that of BPSO-TVMS, BQUATRE, and BGWO-a shows that the difference between the two is not significant on some low-dimensional data sets, with the former only slightly ahead of the latter, e.g., ABBFGO, the best performer in Cancer, is 0.0017 ahead of BGWO-a. It is partly due to the fact that there are only two categories in Cancer, or it may be that the small dimensionality of the data set results in a transfer function with a mutation strategy that does not give the better performance of binary BFGO. Glass has the same number of features as Cancer, but Glass has six categories, resulting in a difference in the accuracy of 0.0138 between ABBFGO-T1 and BPSO-TVMS. However, the difference is more pronounced in the high-dimensional data set, where ABBFGO* is superior, with the best ABBFGO-V2 being 0.0516 ahead of BGWO-a in the Turkish Music Emotion, as well as in the Musk (Version 1), Dnatest, LSVT Voice Rehabilitation and Sonar data sets. The differences were also more pronounced. For the tapered-shaped transfer function, it can be seen in Table 7 that T2 with lower curvature has more advantages than T1 on the high-dimensional data sets of Turkish Music Emotion, Musk (Version 1), Dnatest and LSVT Voice Rehabilitation. From Table 9, it is known that the P-value of the Feldman test is greater than 5% in all 12 data sets, so it can be concluded that there is no significant difference between the algorithms, and the data are considered plausible. Based on the above analysis, it is believed that the transfer function and the long-mutation strategy of the new mutation mode give the binary BFGO stronger performance, which makes it more competitive compared with other advanced algorithms in the high-dimensional multi-type data sets.

## 6. Conclusions

The bamboo forest growth optimization algorithm, inspired by the growth process of bamboo forests, successfully solves many optimization problems. This paper focuses on the analysis of tfs and mutation strategies. Based on the analysed search space and the characteristics of the transfer functions, two different curvatures, V-tfs and T-tfs, are proposed. To avoid the stagnation of the binary BFGO algorithm, the long-mutation strategy with a novel mutation approach is introduced. The newly constructed tfs and the new mutation strategy are tested in 23 benchmark test functions. The experiments show that the newly constructed transfer function has better performance and that the binary BFGO with the long-mutation strategy with a novel mutation has a significant advantage in solving the optimization problem. Feature selection is an important optimization problem and ABBFGO, ABBFGO-S, ABBFGO-V2, ABBFGO-V3, ABBFGO-T1, and ABBFGO-T2 are selected to complete the feature selection and compared with three cutting-edge algorithms, BPSO-TVMS, BQUATRE and BGWO-a. The experiments show that the long-mutation strategy of the transfer function with the new mutation method gives a stronger performance of binary BFGO, and the extent of this improvement is particularly striking on high-dimensional data sets. As this is the first application of BFGO to the discrete domain, much about the capabilities of BFGO has yet to be fully explored and it has more room for development.

## Figures and Tables

**Table 1:** The details of the benchmark functions.

Num	Name	Parameter Space	Dim	Opt
$F_{1}$	Sphere	[−100, 100]	30	0
$F_{2}$	Schwefel’s function 2.21	[−10, 10]	30	0
$F_{3}$	Schwefel’s function 1.2	[−100, 100]	30	0
$F_{4}$	Schwefel’s function 2.221	[−100, 100]	30	0
$F_{5}$	Rosenbrock	[−30, 30]	30	0
$F_{6}$	Step	[−100, 100]	30	0
$F_{7}$	Dejong’s noisy	[−1.28, 1.28]	30	0
$F_{8}$	Schwefel	[−500, 500]	30	−12,569
$F_{9}$	Rastringin	[−5.12, 5.12]	30	0
$F_{10}$	Ackley	[−32, 32]	30	0
$F_{11}$	Griewank	[−600, 600]	30	0
$F_{12}$	Generalized penalized 1	[−50, 50]	30	0
$F_{13}$	Generalized penalized 2	[−50, 50]	30	0
$F_{14}$	Fifth of Dejong	[−65, 65]	2	1
$F_{15}$	Kowalik	[−5, 5]	4	0.0003
$F_{16}$	Six-hump camel back	[−5, 5]	2	−1.0316
$F_{17}$	Branins	[−5, 5]	2	0.398
$F_{18}$	Goldstein–Price	[−2, 2]	2	3
$F_{19}$	Hartman 1	[1, 3]	3	−3.86
$F_{20}$	Hartman 2	[0, 1]	6	−3.32
$F_{21}$	Shekel 1	[0, 10]	4	−10.1532
$F_{22}$	Shekel 2	[0, 10]	4	−10.4028
$F_{23}$	Shekel 3	[0, 10]	4	−10.5363

**Table 2:** The details of the comparison methods.

Algorithm	Transfer Function
BBFGO	Transfer Function from Equation (11)
BBFGO-S	Transfer Function from Equation (12)
BBFGO-V1	Transfer Function from Equation (14)
BBFGO-V2	Transfer Function from Equation (15)
BBFGO-V3	Transfer Function from Equation (16)
BBFGO-T1	Transfer Function from Equation (18)
BBFGO-T2	Transfer Function from Equation (19)
BGWO	Transfer Function from [37]
BPSO-S	Transfer Function from [45]
BQUATRE	Transfer Function from [46]

**Table 3:** Parameter settings of the algorithms used for comparison in the current study.

Algorithm	Parameter	Values
BFGO(BBFGO)	Number of individuals	30
	Maximum number of iterations	500
	Number of bamboo shoots	6
	Number of bamboo whips	5
GWO(BGWO)	Number of wolves	30
	Maximum number of iterations	500
	The *a* parameter	2 * it/MAX_IT
PSO(BPSO-S)	Number of particles	30
	Maximum number of iterations	500
	Inertia weight $w_{max}$	0.9
	Inertia weight $w_{min}$	0.2
	$C_{1}$	2
	$C_{2}$	2
	$V_{min}$, $V_{max}$	[−6, 6]
QUATRE(BQUATRE)	Number of individuals	30
	Maximum number of iterations	500
	Matrix control factor	0.7

**Table 4:** The statistical results of the BBFGO* family algorithms.

Function	BBFGO-S		BBFGO-V1		BBFGO-V2		BBFGO-V3		BBFGO-T1		BBFGO-T2		BBFGO		ABBFGO	
	AVG	STD	AVG	STD	AVG	STD	AVG	STD	AVG	STD	AVG	STD	AVG	STD	AVG	STD
$F_{1}$	0	0	0	0	0	0	0	0	0	0	0	0	4.4667	0.9732	0	0
$F_{2}$	0	0	0	0	0	0	0	0	0	0	0	0	4.9667	0.8503	0	0
$F_{3}$	0	0	0	0	0	0	0	0	0	0	0	0	207.1333	56.8905	0.1	0.5477
$F_{4}$	0	0	0	0	0.1	0.3051	0.0333	0.1826	1	0	0.9333	0.2537	1	0	1	0
$F_{5}$	29	0	29	0	19.3333	13.9044	28.0333	5.2947	1.9333	7.3575	14.9333	31.1392	124.4	82.0431	0	0
$F_{6}$	7.5	0	7.5	0	7.5	0	7.5	0	7.5	0	7.5	0	17.6333	1.5698	7.5	0
$F_{7}$	3.68 × $10^{-5}$	3.75 × $10^{-5}$	3.39 × $10^{-5}$	3.10 × $10^{-5}$	3.23 × $10^{-5}$	2.89 × $10^{-5}$	3.42 × $10^{-5}$	3.13 × $10^{-5}$	3.45 × $10^{-5}$	3.63 × $10^{-5}$	3.56 × $10^{-5}$	4.13 × $10^{-5}$	55.9377	9.5740	4.15 × $10^{-4}$	3.60 × $10^{-4}$
$F_{8}$	−25.2441	1.08 × $10^{-14}$	−21.0648	0.84099	−25.2441	1.08 × $10^{-14}$	−24.6551	0.54802	−25.2441	1.08 × $10^{-14}$	−25.1319	2.91 × $10^{-1}$	−24.6271	0.4908	−25.2441	1.08 × $10^{-14}$
$F_{9}$	0	0	0	0	0	0	0	0	0	0	0	0	4.8667	0.8193	0	0
$F_{10}$	8.88 × $10^{-16}$	0	8.88 × $10^{-16}$	0	8.88 × $10^{-16}$	0	8.88 × $10^{-16}$	0	8.88 × $10^{-16}$	0	8.88 × $10^{-16}$	0	1.5665	0.1383	0.0239	0.1309
$F_{11}$	0	0	0	0	0	0	0	0	0	0	0	0	0.1533	0.0262	0.0017	0.0066
$F_{12}$	1.6690	1.13 × $10^{-15}$	1.6690	1.13 × $10^{-15}$	1.6690	1.13 × $10^{-15}$	1.6690	1.13 × $10^{-15}$	1.6690	1.13 × $10^{-15}$	1.6690	1.13 × $10^{-15}$	2.4223	0.1366	1.6690	1.13 × $10^{-15}$
$F_{13}$	1.35 × $10^{-32}$	5.57 × $10^{-48}$	0.4867	0.1548	1.35 × $10^{-32}$	5.57 × $10^{-48}$	0.0567	0.0568	1.35 × $10^{-32}$	5.57 × $10^{-48}$	0.0400	4.98 × $10^{-2}$	0.0667	0.0547	1.35 × $10^{-32}$	5.57 × $10^{-48}$
$F_{14}$	12.6705	3.61 × $10^{-15}$	12.6705	3.61 × $10^{-15}$	12.6705	3.61 × $10^{-15}$	12.6705	3.61 × $10^{-15}$	12.6705	3.61 × $10^{-15}$	12.6705	3.61 × $10^{-15}$	12.6705	3.61 × $10^{-15}$	12.6705	3.61 × $10^{-15}$
$F_{15}$	0.1484	0	0.1484	0	0.1484	0	0.1484	0	0.1484	0	0.1484	0	0.1484	0	0.1484	0
$F_{16}$	0	0	0	0	0	0	0	0	0	0	0	0	0	0	0	0
$F_{17}$	27.7029	1.81 × $10^{-14}$	27.7029	1.81 × $10^{-14}$	27.7029	1.81 × $10^{-14}$	27.7029	1.81 × $10^{-14}$	27.7029	1.81 × $10^{-14}$	27.7029	1.81 × $10^{-14}$	27.7029	1.81 × $10^{-14}$	27.7029	1.81 × $10^{-14}$
$F_{18}$	600	0	600	0	600	0	600	0	600	0	600	0	600	0	600	0
$F_{19}$	−0.3348	1.69 × $10^{-16}$	−0.3348	1.69 × $10^{-16}$	−0.3348	1.69 × $10^{-16}$	−0.3348	1.69 × $10^{-16}$	−0.3348	1.69 × $10^{-16}$	−0.3348	1.69 × $10^{-16}$	−0.3348	1.69 × $10^{-16}$	−0.3348	1.69 × $10^{-16}$
$F_{20}$	−0.1657	2.82 × $10^{-17}$	−0.1657	2.82 × $10^{-17}$	−0.1657	2.82 × $10^{-17}$	−0.1657	2.82 × $10^{-17}$	−0.1657	2.82 × $10^{-17}$	−0.1657	2.82 × $10^{-17}$	−0.1657	2.82 × $10^{-17}$	−0.1657	2.82 × $10^{-17}$
$F_{21}$	−5.0552	4.52 × $10^{-15}$	−5.0552	4.52 × $10^{-15}$	−5.0552	4.52 × $10^{-15}$	−5.0552	4.52 × $10^{-15}$	−5.0552	4.52 × $10^{-15}$	−5.0552	4.52 × $10^{-15}$	−5.0552	4.52 × $10^{-15}$	−5.0552	4.52 × $10^{-15}$
$F_{22}$	−5.0877	0	−5.0877	0	−5.0877	0	−5.0877	0	−5.0877	0	−5.0877	0	−5.0877	0	−5.0877	0
$F_{23}$	−5.1285	2.71 × $10^{-15}$	−5.1285	2.71 × $10^{-15}$	−5.1285	2.71 × $10^{-15}$	−5.1285	2.71 × $10^{-15}$	−5.1285	2.71 × $10^{-15}$	−4.9891	7.63 × $10^{-1}$	−5.1285	2.71 × $10^{-15}$	−5.1285	2.71 × $10^{-15}$

**Table 5:** The statistical results of BQUATRE, BGWO-a and BPSO-TVMS.

Function	BQUATRE		BGWO-a		BPSO-TVMS	
	AVG	STD	AVG	STD	AVG	STD
$F_{1}$	0	0	2.8333	1.2058	1.1667	0.5921
$F_{2}$	0.0333	0.1826	3.1667	1.5555	1.4333	0.6789
$F_{3}$	0.4667	1.1059	83.7667	67.5174	17.1333	13.9599
$F_{4}$	1	0	1	0	1	0
$F_{5}$	0	0	0	0	93.5667	90.9607
$F_{6}$	7.5	0	13.7667	2.5587	10.4333	1.4606
$F_{7}$	2.6001	3.6540	39.0334	21.0557	9.1307	4.5008
$F_{8}$	−25.2441	1.08 × $10^{-14}$	−25.2441	1.08 × $10^{-14}$	−24.0100	0.4808
$F_{9}$	0	0	3.5	1.3326	1.4333	0.5040
$F_{10}$	8.88 × $10^{-16}$	0	1.1964	0.3619	0.8040	0.1349
$F_{11}$	0.0144	0.0187	0.1390	0.0572	0.0293	0.0154
$F_{12}$	1.6725	1.33 × $10^{-2}$	2.1409	0.2106	1.8653	0.0981
$F_{13}$	1.35 × $10^{-32}$	5.57 × $10^{-48}$	1.35 × $10^{-32}$	5.57 × $10^{-48}$	0.1500	0.0509
$F_{14}$	12.6705	3.61 × $10^{-15}$	12.6705	3.61 × $10^{-15}$	12.6705	3.61 × $10^{-15}$
$F_{15}$	0.1484	0	0.1484	0	0.1484	0
$F_{16}$	0	0	0	0	0	0
$F_{17}$	27.7029	1.81 × $10^{-14}$	27.7029	1.81 × $10^{-14}$	27.7029	1.81 × $10^{-14}$
$F_{18}$	600	0	600	0	600	0
$F_{19}$	−0.3348	1.69 × $10^{-16}$	−0.3337	0.0063	−0.3348	1.69 × $10^{-16}$
$F_{20}$	−0.1657	2.82 × $10^{-17}$	−0.1397	0.0479	−0.1657	2.82 × $10^{-17}$
$F_{21}$	−5.0552	4.52 × $10^{-15}$	−5.0552	4.52 × $10^{-15}$	−5.0552	4.52 × $10^{-15}$
$F_{22}$	−5.0877	0	−5.0877	0	−5.0877	0
$F_{23}$	−5.1285	2.71 × $10^{-15}$	−5.1285	2.71 × $10^{-15}$	−5.1285	2.71 × $10^{-15}$

**Table 6:** The details of the testing data sets.

Dataset	No. of Features	No. of Instances	No. of Classes
Turkish Music Emotion	50	400	4
Musk (Version 1)	166	476	2
Cancer	9	683	2
Dermatology	34	366	6
Dnatest	180	1186	3
German	24	1000	4
Glass	9	214	6
Heartstatlog	13	270	2
Ionosphere	34	351	2
LSVT Voice Rehabilitation	310	126	2
Sonar	60	208	2
WDBC	30	569	2

**Table 7:** The accuracy and number of the compared algorithms.

Dataset	ABBFGO		ABBFGO-S		ABBFGO-V2		ABBFGO-V3		ABBFGO-T1		ABBFGO-T2		BPSO-TVMS		BQUATRE		BGWO-a	
	Acc	Num	Acc	Num	Acc	Num	Acc	Num	Acc	Num	Acc	Num	Acc	Num	Acc	Num	Acc	Num
Turkish Music Emotion	0.7617	25.67	0.7748	11.80	**0.7902**	17.53	0.7879	16.73	0.7783	20.40	0.7881	15.87	0.7098	22.33	0.7005	38.27	0.7386	27.40
Cancer	**0.9787**	5.67	0.9775	4.80	0.9766	4.27	0.9766	4.33	0.9768	4.47	0.9770	4.60	0.9766	5.40	0.9720	6.07	0.9770	5.80
Musk (Version 1)	0.9149	85.93	0.9318	46.27	**0.9424**	47.20	0.9331	43.53	0.9286	58.00	0.9420	49.33	0.8786	81.73	0.8627	136.67	0.9073	86.87
Dermatology	0.9904	19.13	0.9847	15.60	0.9904	18.27	0.9909	18.00	**0.9912**	20.13	0.9910	19.53	0.9802	19.40	0.9789	25.87	0.9854	21.53
Dnatest	0.8548	91.07	0.9041	27.53	**0.9092**	16.80	0.9074	17.67	0.8924	39.33	0.9057	20.53	0.8191	85.40	0.8044	132.80	0.8564	90.47
German	0.5076	12.93	0.5038	6.80	0.5121	9.60	0.5070	7.33	**0.5134**	12.67	0.5107	10.13	0.4886	11.07	0.4752	16.53	0.4980	13.53
Glass	0.4559	3.60	0.4521	2.93	0.4610	3.73	0.4609	3.80	**0.4616**	4.07	0.4559	3.47	0.4478	3.80	0.3748	4.80	0.4413	4.73
Heartstatlog	0.8562	5.20	0.8569	3.87	0.8588	4.33	0.8580	5.13	**0.8608**	5.87	0.8562	4.80	0.8494	4.60	0.8292	7.80	0.8440	7.13
Ionosphere	0.9291	10.13	0.9387	5.20	**0.9415**	4.47	0.9377	4.33	0.9385	7.73	0.9409	4.27	0.9066	11.27	0.9023	15.80	0.9168	13.33
LSVT Voice Rehabilitation	0.9192	154.87	0.9421	50.67	0.9462	43.27	**0.9482**	29.40	0.9310	82.87	0.9405	45.87	0.8717	151.20	0.8234	231.47	0.9073	149.73
Sonar	0.9081	27.93	0.9125	14.67	**0.9321**	16.53	0.9257	15.27	0.9290	23.00	0.9238	17.40	0.8600	27.40	0.8431	42.07	0.9023	30.60
WDBC	0.9851	14.80	0.9843	7.60	**0.9853**	9.13	0.9846	8.13	0.9849	10.33	0.9848	8.53	0.9831	13.87	0.9739	21.73	0.9838	14.47

If an algorithm has the best classification accuracy on the data set, its data are marked as bold.

**Table 8:** The fitness of the compared algorithms.

Dataset	ABBFGO		ABBFGO-S		ABBFGO-V2		ABBFGO-V3		ABBFGO-T1		ABBFGO-T2		BPSO-TVMS		BQUATRE		BGWO-a	
	Fitness	Rank	Fitness	Rank	Fitness	Rank	Fitness	Rank	Fitness	Rank	Fitness	Rank	Fitness	Rank	Fitness	Rank	Fitness	Rank
Turkish Music Emotion	0.2412	6	0.2254	5	0.2112	1	0.2134	3	0.2236	4	0.2130	2	0.2919	9	0.2546	7	0.2644	8
Cancer	0.0274	1	0.0276	2	0.0279	4	0.0280	5	0.0280	5	0.0278	3	0.0292	7	0.0284	6	0.0292	7
Musk (Version 1)	0.0895	6	0.0703	4	0.0599	1	0.0688	3	0.0742	5	0.0604	2	0.1251	9	0.1151	8	0.0970	7
Dermatology	0.0151	4	0.0197	5	0.0148	3	0.0143	1	0.0147	2	0.0147	2	0.0253	7	0.0151	4	0.0208	6
Dnatest	0.1488	7	0.0965	4	0.0909	1	0.0927	2	0.1087	5	0.0945	3	0.1838	9	0.1769	8	0.1472	6
German	0.4928	5	0.4941	6	0.4870	1	0.4912	3	0.4870	1	0.4887	2	0.5109	8	0.4926	4	0.5026	7
Glass	0.5427	5	0.5457	7	0.5378	2	0.5379	3	0.5375	1	0.5425	4	0.5509	8	0.5433	6	0.5584	9
Heartstatlog	0.1463	6	0.1447	4	0.1431	2	0.1446	3	0.1423	1	0.1460	5	0.1527	8	0.1484	7	0.1600	9
Ionosphere	0.0732	6	0.0622	3	0.0593	1	0.0629	4	0.0632	5	0.0598	2	0.0958	9	0.0790	7	0.0863	8
LSVT Voice Rehabilitation	0.0850	6	0.0589	3	0.0547	2	0.0522	1	0.0710	5	0.0604	4	0.1319	9	0.1291	8	0.0966	7
Sonar	0.0957	6	0.0891	5	0.0700	1	0.0762	3	0.0741	2	0.0783	4	0.1431	9	0.1158	8	0.1019	7
WDBC	0.0197	6	0.0181	4	0.0176	1	0.0179	3	0.0184	5	0.0179	2	0.0214	9	0.0206	7	0.0209	8
Total	64		52		20		34		41		35		101		80		89	

**Table 9:** The result of Friedman test on feature selection.

Dataset	Sum of Squares	Degree of Freedom	Mean Squares	*p*-Value
Turkish Music Emotion	2845.41	140	20.3244	0.6354
Cancer	2876.36	140	20.5455	0.9315
Musk (Version 1)	2798.73	140	19.9909	0.4450
Dermatology	2751.55	140	19.6539	0.3303
Dnatest	2741.45	140	19.5818	0.2625
German	2797.09	140	19.9792	0.4440
Glass	2743.91	140	19.5994	0.2792
Heartstatlog	2939.45	140	20.9961	0.9499
Ionosphere	2772.23	140	19.8016	0.3581
LSVT Voice Rehabilitation	2852.50	140	20.3750	0.6619
Sonar	2851.45	140	20.3675	0.6540
WDBC	2812.00	140	20.0857	0.5548

## Data Availability

The data involved in this study are all public data, which can be found here: http://archive.ics.uci.edu/ml/index.php (accessed on 5 August 2022).
